# UV Screening in Native and Non-native Plant Species in the Tropical Alpine: Implications for Climate Change-Driven Migration of Species to Higher Elevations

**DOI:** 10.3389/fpls.2017.01451

**Published:** 2017-08-22

**Authors:** Paul W. Barnes, Ronald J. Ryel, Stephan D. Flint

**Affiliations:** ^1^Department of Biological Sciences and Environment Program, Loyola University New Orleans, New Orleans LA, United States; ^2^Department of Wildland Resources, Utah State University, Logan UT, United States; ^3^Department of Forest, Rangeland and Fire Sciences, University of Idaho, Moscow ID, United States

**Keywords:** alpine, elevation gradient, epidermal UV-A transmittance, flavonoids, Hawaii, native species, non-native species, ultraviolet radiation

## Abstract

Ongoing changes in Earth’s climate are shifting the elevation ranges of many plant species with non-native species often experiencing greater expansion into higher elevations than native species. These climate change-induced shifts in distributions inevitably expose plants to novel biotic and abiotic environments, including altered solar ultraviolet (UV)-B (280–315 nm) radiation regimes. Do the greater migration potentials of non-native species into higher elevations imply that they have more effective UV-protective mechanisms than native species? In this study, we surveyed leaf epidermal UV-A transmittance (T_UV A_) in a diversity of plant species representing different growth forms to test whether native and non-native species growing above 2800 m elevation on Mauna Kea, Hawaii differed in their UV screening capabilities. We further compared the degree to which T_UV A_ varied along an elevation gradient in the native shrub *Vaccinium reticulatum* and the introduced forb *Verbascum thapsus* to evaluate whether these species differed in their abilities to adjust their levels of UV screening in response to elevation changes in UV-B. For plants growing in the Mauna Kea alpine/upper subalpine, we found that adaxial T_UV A_, measured with a UVA-PAM fluorometer, varied significantly among species but did not differ between native (mean = 6.0%; *n* = 8) and non-native (mean = 5.8%; *n* = 11) species. When data were pooled across native and non-native taxa, we also found no significant effect of growth form on T_UV A_, though woody plants (shrubs and trees) were represented solely by native species whereas herbaceous growth forms (grasses and forbs) were dominated by non-native species. Along an elevation gradient spanning 2600–3800 m, T_UV A_ was variable (mean range = 6.0–11.2%) and strongly correlated with elevation and relative biologically effective UV-B in the exotic *V. thapsus*; however, T_UV A_ was consistently low (3%) and did not vary with elevation in the native *V. reticulatum*. Results indicate that high levels of UV protection occur in both native and non-native species in this high UV-B tropical alpine environment, and that flexibility in UV screening is a mechanism employed by some, but not all species to cope with varying solar UV-B exposures along elevation gradients.

## Introduction

Many plant species are migrating in response to ongoing changes in climate and additional shifts in geographic ranges are expected in the future, though the rates of movement will likely vary substantially with growth form (e.g., herbaceous vs. woody plants; IPCC, 2014). For species in montane environments, recent climate change-induced shifts in species distributions toward higher elevations have been documented in temperate and tropical locations (Benavides et al., 2016; Dolezal et al., 2016). Over the past 100–200 years, many non-native (i.e., introduced or alien) species have colonized high altitude environments (Pyšek et al., 2011) and in several temperate mountain ranges in North America and Europe, non-native species appear to be migrating to higher elevations to a greater degree than native species (Wolf et al., 2016; Dainese et al., 2017). These findings suggest that, at least along elevation gradients, non-native species have higher migration potentials than native species, though this may depend upon levels of disturbance and local habitat heterogeneity (Suding et al., 2015; Averett et al., 2016). This upward migration of species inevitably exposes plants to novel combinations of biotic and abiotic environmental conditions, including ultraviolet (UV) radiation (280–400 nm), with the potential for significant negative impacts on native alpine biodiversity (Chapin and Körner, 1994; Savage and Vellend, 2015; Cuyckens et al., 2016).

Because of differences in atmospheric conditions (primarily optical depth of the atmosphere and the thickness of the stratospheric ozone layer) and prevailing solar angles, the levels of solar UV-B radiation (280–315 nm) generally increase with decreasing latitude and increasing altitude (Caldwell et al., 1980; Blumthaler et al., 1997; McKenzie et al., 2001). Consequentially, tropical alpine environments experience some of the highest UV-B irradiances on the Earth’s surface. UV-B radiation is known to induce a number of potentially deleterious effects in plants, including disruption of the integrity and function of important macromolecules (DNA, proteins, and lipids), oxidative damage, partial inhibition of photosynthesis and growth reduction (Albert et al., 2011; Jansen and Bornman, 2012; Hideg et al., 2013). However, plants have evolved photosensory mechanisms to detect UV (Tilbrook et al., 2013; Jenkins, 2014) and then protect and repair sensitive targets from direct and indirect UV-induced injury (Jansen et al., 1998; Britt, 1999) such that the negative effects of ambient UV-B on plant growth and productivity are typically small or difficult to detect under field conditions (Ballaré et al., 2011). Nonetheless, UV-B is generally considered to be an important selective force in the evolution and adaptation of the tropical alpine flora (Lee and Lowry, 1980; Robberecht et al., 1980; Caldwell et al., 1982). To what extent UV-B limits the ability of plant species to migrate into alpine environments or expand their ranges within the alpine, however, is not known.

One of the most important and widespread protective responses of plants to UV radiation involves the induction and synthesis of flavonoids, hydroxycinnamic acids (HCAs) and related phenylpropanoids that function as “UV sunscreens” and antioxidants (Searles et al., 2001; Agati et al., 2012; Schreiner et al., 2012). Flavonoid biosynthesis is influenced by UV-B, UV-A (315–400 nm), and visible radiation (400–700 nm) (Flint et al., 2004; Siipola et al., 2015) and appears to be mediated, at least in part, by the UV-B photoreceptor UV RESISTANCE LOCUS 8 (UVR8) (Morales et al., 2013). The accumulation of flavonoids and related UV-absorbing compounds in epidermal tissue decreases epidermal UV transmittance (Mazza et al., 2000; Bidel et al., 2007) and is a primary mechanism by which plants acclimate to changing UV environments, including alterations resulting from stratospheric ozone depletion and climate change (Caldwell et al., 1983; Bornman et al., 2015).

This UV screening response entails measurable energetic and growth costs (Snell et al., 2009; Hofmann and Jahufer, 2011) and varies within and among plant species (e.g., Qi et al., 2010; Randriamanana et al., 2015). Some of the interspecific variation in UV screening can be attributable to growth form differences in leaf structure and cellular distributions of UV-absorbing compounds (i.e., vacuole vs. cell wall; Day et al., 1992, 1993). For example, in a study using micro-probes to measure UV penetration into the foliage of a diverse group of plants in the North American Rocky Mountains, Day et al. (1992) found that the leaf epidermis of herbaceous dicots (forbs) was less effective in attenuating UV-B than that of grasses and woody dicots. The accumulation of UV-absorbing compounds and resultant changes in leaf optical properties are also highly plastic traits in many species (Wargent et al., 2015) and have been shown to vary in relation to natural elevation/latitudinal UV-B gradients (Robberecht et al., 1980; Rozema et al., 1997; Ruhland et al., 2013). In many cases, these differences in UV protection can account, at least in part, for the differential UV-B sensitivities of high- vs. low-elevation taxa (Barnes et al., 1987; Sullivan et al., 1992; Ziska et al., 1992; but see Nybakken et al., 2004).

At present, very little is known whether native and non-native plant species differ in their tolerances to UV-B and levels of UV protection. The apparent greater propensity for non-native species to migrate to higher elevations than native species may indicate that non-native species are capable of adjusting their UV protection more effectively, either through greater phenotypic plasticity or more rapid genetic adaptation, than native species. Indeed, the success of non-native species in general is often attributed, in part, to their high degree of phenotypic plasticity to environmental change (Richards et al., 2006; Davidson et al., 2011). If non-native species exhibit greater phenotypic plasticity to UV-B change than native species, one would expect greater variation in UV protective mechanisms along elevational/UV-B gradients relative to native species, and non-native species would display similar or perhaps even higher levels of UV protection than native species in the high UV-B alpine environment. Alternatively, many non-native species possess functional traits (physiology, allocation, growth rate, etc.) that allow for high rates of resource acquisition and performance (Van Kleunen et al., 2010; Funk, 2013) and these traits can come at a cost in reduced tolerance to abiotic stress in harsh conditions, such as occurs in the alpine (Brock and Galen, 2005). Tolerance to UV-B is often cross-linked with tolerance to other abiotic stresses such as drought (Bandurska et al., 2013) and defense against pests and pathogens (Mewis et al., 2012; Zavala et al., 2015). It is thus conceivable that non-native species may invest less in UV protection than native species and may be more rather than less sensitive to UV-B than native species. If this is the case, the invasion of high elevation habitats by non-native species may be governed less by UV-B tolerance than by other plant attributes, such as life history traits and competitive abilities. In support of this hypothesis, Wang H. et al. (2016) reported that non-native populations of *Triadica sebifera* exhibited greater sensitivity to elevated UV-B than native populations under controlled conditions. Whether these differences in UV tolerance were the result of differences in UV-absorbing compounds and UV screening was not investigated. Taken together it is therefore difficult to predict, *a priori*, whether non-native species that occur in high elevation environments would exhibit similar, higher or lower levels of UV protection than their native counterparts.

In the study described here, we characterize the leaf optical properties of a suite of native and non-native plant species of different growth forms growing in the tropical alpine and upper subalpine of Mauna Kea, Hawaii to test whether differences in epidermal UV transmittance (measured as the screening of UV-A radiation) exist between native vs. non-native species. Additionally, we examine UV screening of a native and a non-native species along an elevation gradient spanning 2600–3800 m to determine if these species differ in their abilities to adjust their levels of UV protection in response to natural variation in UV-B exposure. This study examines the ability of native and non-native plant species to cope with extreme natural levels of UV-B, and thus provides insights into the role that UV-B may play in influencing climate change-induced upward range expansions in mountains.

## Materials and Methods

### Survey of Native and Non-native Alpine Species

Studies were conducted on 19 common native (8) and non-native (11) wild species growing on native volcanic soil in un-shaded habitats on the south slope of Mauna Kea, Hawaii, United States (19°45′N, 155°27′W) ca. 2800–3900 masl from early to mid-June (**Table 1**). This elevation range includes the upper subalpine and alpine vegetation zones of Mauna Kea (Gerrish, 2013). For all species, we sampled plants that were growing at, or near, their approximate peak elevations as determined from floristic surveys (**Table 1**; Wagner et al., 1999). We were limited in the amount of the Mauna Kea alpine/upper subalpine vegetation we could sample in due to logistical constraints (i.e., there are very few roads on the mountain) and out of deference to the indigenous Hawaiian culture that considers the mountain to be sacred. The majority of our sampling was therefore conducted within the south-facing slope of the 212 km^2^ Mauna Kea Forest Reserve, including sites along the Mauna Kea access road leading from the Mauna Kea Information Visitor’s Center (2800 masl) to the summit (ca. 4200 masl). Within this area, we selected species for study that were easily accessible (they occurred within ca. 1 km of the Mauna Kea Access Road), relatively abundant (at least 10 individuals present per sampling location), and which were suitable for measurement (large enough leaves to fit the sampling chamber and green in color; see below).

**Table 1:** Characteristics of the native and non-native (introduced) species sampled for epidermal UV-A transmittance in the Mauna Kea, Hawaii alpine/subalpine.

Species	Family	Growth form	Elevation sampled (m)	Elevation range (m)
**Native species**				
*Leptecophylla tameiameiae*	Epacridaceae	Shrub	3429	60–3230
*Chenopodium oahuense*	Amaranthaceae	Shrub	2774	0–2520
*Geranium cuneatum*	Geraniaceae	Shrub	3444	1480–3250
*Sophora chrysophylla*	Fabaceae	Shrub/tree	2914	450–3240
*Vaccinium reticulatum*	Ericaceae	Shrub	3353	640–3700
*Stenogyne microphylla*	Lamiaceae	Vine/forb	2774	1200–2700
*Cystopteris douglasii*	Woodsiaceae	Fern	3962	1500–3000+
*Trisetum glomeratum*	Poaceae	Grass	3962	750–4090
**Non-native species**				
*Malva parviflora*	Malvaceae	Forb	2774	0–2270
*Verbascum thapsus*	Scrophulariaceae	Forb	3962	1550–2350
*Taraxacum officinale*	Asteraceae	Forb	3962	NA
*Oenothera stricta*	Onagraceae	Forb	2774	1200–2740
*Heterotheca grandiflora*	Asteraceae	Forb	2774	10–2270
*Verbascum virgatum*	Scrophulariaceae	Forb	2774	NA
*Rumex acetosella*	Polygonaceae	Forb	3962	1115–2840
*Hypochaeris radicata*	Asteraceae	Forb	3429	1100–2800
*Senecio madagascariensis*	Asteraceae	Forb	2914	NA
*Poa pratensis*	Poaceae	Grass	3962	1220–4025
*Anthoxanthum odoratum*	Poaceae	Grass	2914	840–2140

*Nomenclature, native vs. non-native classifications, growth form and elevation ranges are from Wagner et al. (1999) and Palmer (2003), according to recent updates. NA, not available.*

The primary goal of this study was to compare UV screening in native and non-native species growing in this tropical alpine environment. We recognized, however, that there was a diversity of plant growth forms in the Mauna Kea alpine, and results from previous studies (e.g., Day et al., 1992) have shown that UV screening can vary significantly among plant growth forms. Thus, we also wished to compare UV screening among plant growth forms to determine if any potential differences in UV screening between native and non-native species could be attributable to growth form differences. We attempted to survey species representing all of the major growth forms present on Mauna Kea [i.e., woody dicots (trees and shrubs), herbaceous dicots (forbs), and grasses]. We did not examine any conifers (none occurred at our study site) and we also did not sample any cushion plants, rosettes or succulent growth forms that are often found in alpine life zones (Körner, 2003) but which are rare or absent in the Mauna Kea alpine (Gerrish, 2013). As a consequence of the sampling limitations described above, the native species tested were mostly woody species (i.e., five of the eight species were shrubs or trees) whereas all of the non-native species were herbs (forbs or grasses; **Table 1**). There are no non-native woody species in the Mauna Kea alpine (Gerrish, 2013). Thus, plant growth form in this study is, to a certain degree, inherently confounded with native vs. non-native status. Also, the native species sampled were all perennials, whereas the non-natives included both annuals and perennials. One species of native fern was sampled. Nomenclature follows Wagner et al. (1999) for the angiosperms and Palmer (2003) for the fern as per recent updates by Wagner et al. (2012).

Measurements of UV screening [epidermal UV-A transmittance (T_UV A_); see below] were taken on 10 plants/species selected haphazardly at each sampling location with two to three leaves measured per plant. There was no systematic pattern of leaf sampling within each plant (i.e., we made no attempt to isolate the effect of leaf position or age on T_UV A_). Rather, we haphazardly selected several leaves among the healthy, mature leaves on an individual plant shoot. Preliminary analyses (ANOVA) showed no significant effect of leaf sample number on T_UV A_. Thus, data were averaged within a plant and subjected to an arcsine transformation (Zar, 1999) to normalize data prior to statistical analysis. We used individual one-way ANOVAs (SAS JMP, Cary, NC, United States) to test for species, growth form, and native vs. non-native effects. In the ANOVA testing for the effect of species on T_UV A_, the individual plant was the unit of replication. For the other ANOVAs (growth form and native vs. non-native comparisons) we averaged values within a species such that the individual species was the unit of replication. Significant differences were determined at *P* < 0.05.

### Elevation Gradient Study

One native and non-native species were selected for additional study to explore whether UV screening varied with elevation and prevailing levels of solar radiation. For this study, we sampled the native shrub, *Vaccinium reticulatum*, and the non-native forb, *Verbascum thapsus*, across the entire elevational range of both species (762–3352 masl for *V. reticulatum*; 100–3962 masl for *V. thapsus*) during June. We chose these species because they could often be found growing in close proximity throughout much of this elevation gradient, which was essential for sampling purposes (measurements were conducted in the dark as indicated below). Sampling locations were located on the south slope of Mauna Kea along a transect that generally corresponded to that used by Nullet and Juvik (1997) in a study characterizing elevation changes in UV-B, photosynthetically active radiation (PAR; 400–700 nm), and total shortwave (SW) radiation (300–3000 nm). In their study, Nullet and Juvik (1997) measured UV-B using a broadband sensor (Robertson-Berger Model 501A Biometer) that provided a measure of biologically effective UV-B (UV-B_ERY_) weighted according to the human erythemal action spectrum. PAR was measured with a LiCor Model LI190SB quantum sensor and SW radiation was measured with an Eppley PSP pyranometer. Nullet and Juvik (1997) collected radiation data at 10 elevations ranging from sea level (0 m) to 4230 masl on Mauna Kea near midday under clear skies in June and then they adjusted their data to correspond to a solar zenith angle of 10°. At each of their sampling elevations, these investigators reported values of UV-B_ERY_, PAR, and SW relative to those at the Mauna Kea summit. We used polynomial regression models [second-order for UV-B and fourth-order for PAR and SW (*R*^2^ = 0.98–0.99); SAS JMP] to establish relationships between elevation and these three measures of relative solar irradiance. We then used these regression models to calculate relative clear sky UV-B_ERY_, PAR, and SW for the sampling elevations used in our study. Least square regression and correlation (multivariate) analyses in JMP were used to examine relationships between T_UV A_, elevation, and solar radiation. For these regression models we tested linear and polynomial models (second, third, and fourth order) and selected the model that explained the largest amount of variation in the data (i.e., the highest value of *R*^2^).

### Measurements of Leaf Optical Properties

Non-invasive measurements of epidermal T_UV A_ were made on adaxial (upper) surfaces of healthy, fully expanded leaves with a field-portable pulse amplitude modulation (PAM) chlorophyll fluorometer (UVA-PAM; Gademann Instruments, Würzburg, Germany). This instrument provides estimates of epidermal T_UV A_ by measuring the fluorescence yield of chlorophyll (*F*_o_; λ > 650 nm) induced by UV-A (375 nm) and blue (BL; 470 nm) radiation, as outlined by Kolb et al. (2005) and following the precautions and procedures of Barnes et al. (2008). This technique is based on the premise that both UV-A and BL can induce chlorophyll fluorescence and that reductions in the penetration of UV to the mesophyll (e.g., from UV-absorbing compounds in the epidermis) will reduce UV-A-induced chlorophyll fluorescence (*F*_UV A_). Fluorescence induced by BL (*F*_BL_), which is not absorbed by UV pigments, serves as a reference to account for variation in chlorophyll content and distribution in the mesophyll. Ideally, calculations of epidermal UV transmittance using this technique are based on the *F*_UV A_/*F*_BL_ of epidermis-free leaf samples. As it is usually not possible to readily remove the epidermis for most species, *F*_UV A_/*F*_BL_ values are normally expressed relative to a blue plastic standard (Heinz Walz GmbH, Effeltrich, Germany), which has emission properties similar to an epidermis-free green leaf. Such was the case in this study. The epidermal T_UV A_ reported here should therefore be considered as approximations of the true transmittances for these species.

Measurements of T_UV A_ by the UVA-PAM generally exhibit strong, positive correlations with direct measurements of epidermal UV transmittance (in both the UV-B and UV-A) made from epidermal peels (Markstadter et al., 2001), and this technique has been widely used to investigate UV sunscreen protection in a diversity of plant species and conditions (see reviews of Barnes et al., 2015; Julkunen-Tiitto et al., 2015; and references therein). However, while measurements of T_UV A_ made by the UVA-PAM are generally correlated with epidermal UV-B transmittances (T_UV B_), the specific relationships between T_UV B_ and T_UV A_ can vary with species, depending on the type of UV-absorbing compounds employed (e.g., flavonoids vs. HCAs; Bilger et al., 2001). Thus, while epidermal T_UV A_ measurements made with the UVA-PAM can serve as reasonable estimates of the overall UV screening of leaves (including T_UV B_, which is technically much more difficult to measure in the field than T_UV A_), we are unable to precisely relate levels of UV-A screening to that for UV-B in the species surveyed in this study. Also, the presence of anthocyanins in the epidermis can introduce errors in determining T_UV A_ with the UVA-PAM by affecting the penetration of the reference (*F*_BL_) beam (Barnes et al., 2000; Pfündel et al., 2007). To avoid these errors, we restricted our sampling to include only plants with green leaves (i.e., leaves that had no visible reddish coloration which would be indicative of anthocyanin accumulation).

Previously, we reported that several of our study species (*V. thapsus* and *Oenothera stricta*) exhibited diurnal changes in epidermal T_UV A_ at this study location, with absolute values of T_UV A_ decreasing 1–3% from predawn to midday and then increasing to predawn levels at sunset (Barnes et al., 2008). Although diurnal changes in T_UV A_ are now known to occur in many species (Barnes et al., 2016a), it is unknown if all the species studied here undergo these diurnal changes. To allow for comparisons among species in the alpine survey we therefore measured T_UV A_ of all species under clear skies during midday (10:00 to 14:00 h local time). These values thus represent maximum levels of UV screening (minimum T_UV A_) for all species regardless of whether or not they adjust T_UV A_ throughout the day. For the elevation study, we did not want diurnal changes in T_UV A_, which could potentially vary in magnitude with temperature and sky conditions (Barnes et al., 2016a,b), to confound elevation/UV-B effects on UV screening. For this study, we therefore measured T_UV A_ ca. 1 h prior to sunrise (predawn). These values thus represent the “baseline” level of UV screening within each species.

## Results

### Survey of Native and Non-native Alpine Species

**FIGURE 1:**
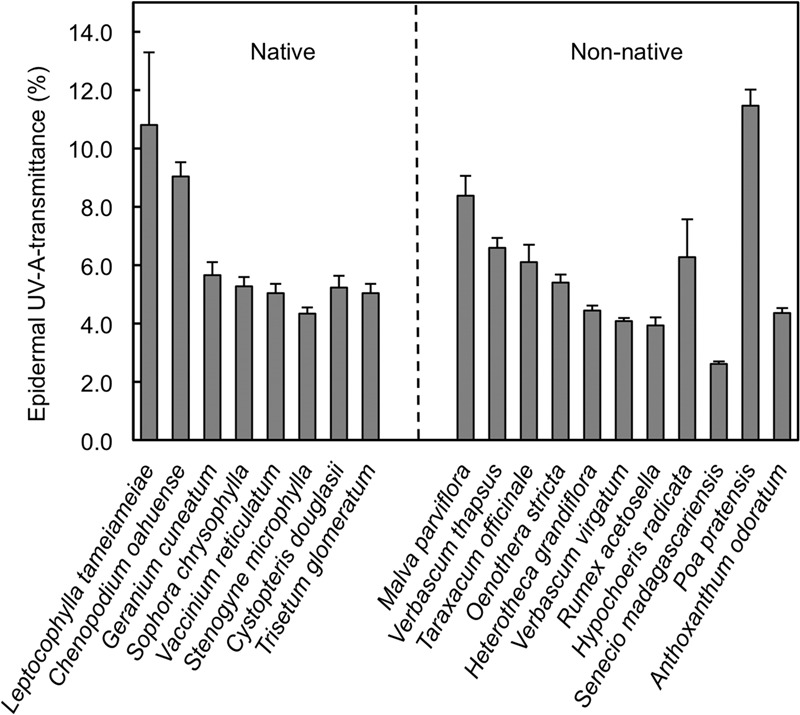
Midday adaxial epidermal UV-A transmittance in native and non-native plant species in the alpine and upper subalpine zones of Mauna Kea, Hawaii. Data are means + SE (*n* = 10). The order of species reflects that in **Table 1** and is based, generally, on similarity in growth form.

**FIGURE 2:**
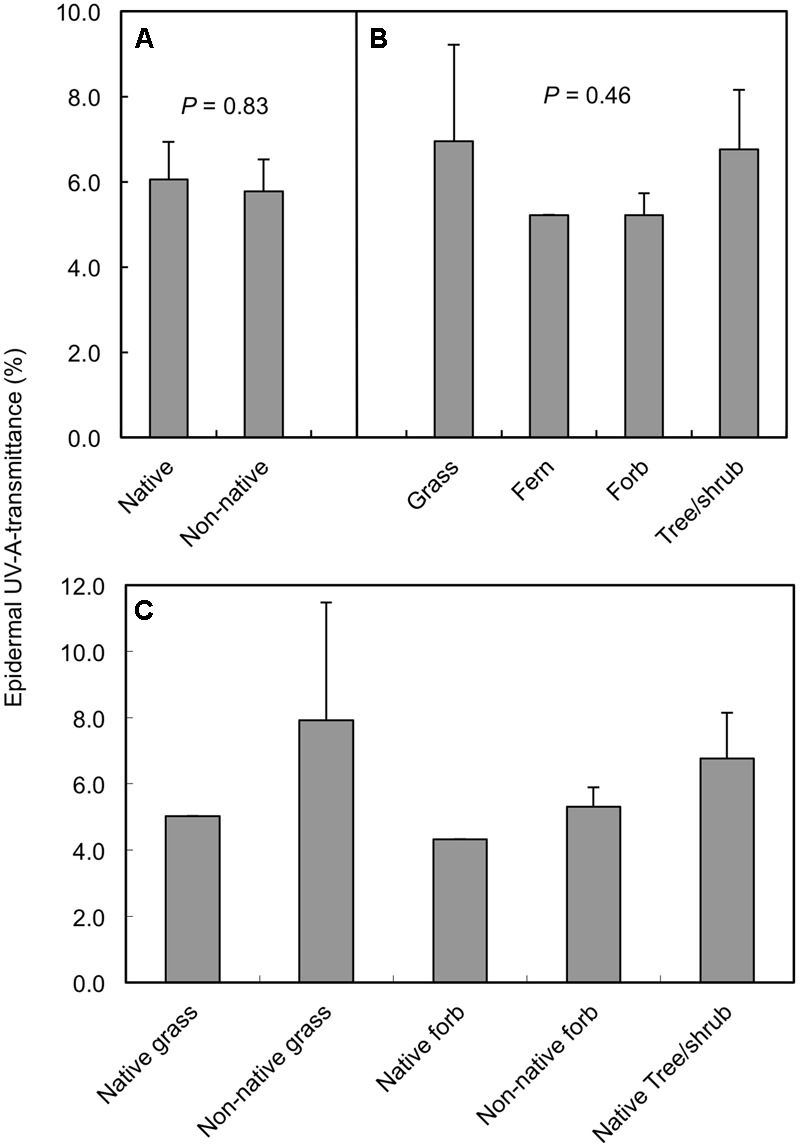
Mean midday adaxial epidermal UV-A transmittance of plant species in the alpine and upper subalpine zones of Mauna Kea, Hawaii grouped according to native vs. non-native status **(A)**, growth form **(B)**, and growth forms within native and non-native categories **(C)**. Data are means + SE with *n* = 8 and 11 for native and non-native species in panel **(A)**; *n* = 3, 1, 10, and 5 for grass, fern, forb, and tree/shrub growth forms, respectively in panel **(B)**; and *n* = 1–5 in panel **(C)** (see **Table 1** for additional information). *P* values for the effect of native vs. non-native status **(A)** and growth form **(B)** are from individual one-way ANOVAs; the one fern species was not included in the growth form ANOVA.

Significant variation in daily minimum epidermal T_UV A_ existed among the plant species measured in the alpine/upper subalpine on Mauna Kea (**Figure 1**; ANOVA, *F*_18,79_ = 14.8; *P* < 0.0001). Mean values of T_UV A_ at midday ranged from a low of 2.6% in *Senecio madagascariensis* to a high of 11.5% in *Poa pratensis* (**Figure 1**). However, when averaged at the species level we detected no significant difference (ANOVA; *F*_1,17_ = 0.05; *P* = 0.83) between native and non-native taxa (**Figure 2A**). Similarly, we found no significant effect (ANOVA; *F*_2,15_ = 0.82; *P* = 0.46) of growth form on T_UV A_, when averaged at the level of species (**Figure 2B**). However, data were variable and forbs represented a disproportionate fraction of the species tested (*n* = 10) as compared to grasses (*n* = 3) and woody plants (trees and shrubs; *n* = 5). The single fern species was excluded from the growth form analysis. We found no consistent patterns in T_UV A_ in growth forms between native and non-native species but replication was insufficient to test for statistical differences (**Figure 2C**). Even though measurements were taken over a range of elevations (2774–3962 masl), we found no significant relationship between T_UV A_ and elevation for the species sampled (*r* = 0.20; *P* = 0.42; not shown). The average sampling elevation of native species (3326 masl) was also not significantly different (ANOVA; *F*_1,17_ = 0.02; *P* = 0.89) than that for the non-native species (3291 masl).

### Elevation Gradient Study

**FIGURE 3:**
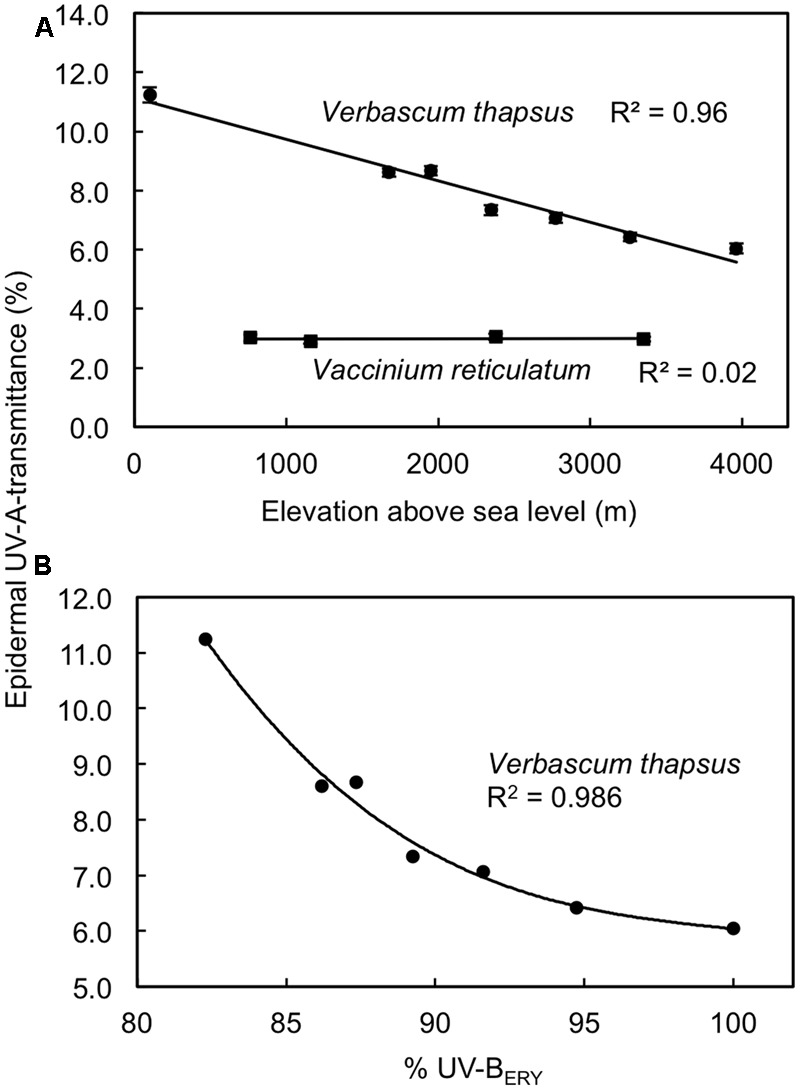
Relationships between elevation and predawn adaxial epidermal UV-A transmittances in *Vaccinium reticulatum* (native species) and *Verbascum thapsus* (non-native species) **(A)**, and the relationship between midday summer clear sky erythemal UV-B irradiance (UV-B_ERY_) and predawn adaxial epidermal UV-A transmittance in *V. thapsus* **(B)**. Data are means ± SE (*n* = 10). Linear regression models in panel **(A)** were significant for *V. thapsus* (*P* < 0.001; *y* = 11.13–0.0014*x*) but not for *V. reticulatum* (*P* = 0.87). The best-fit line relating epidermal UV-A transmittance to %UV-B_ERY_ for *V. thapsus* in panel **(B)** is a third-order polynomial model [*y* = 32.2 – 0.28*x* + 0.022(*x* – 90.2)^2^ – 0.0007(*x* – 90.2)^3^], which is significant at *P* = 0.003. UV-B_ERY_ data are expressed relative to the value at the highest sampling elevation on Mauna Kea as per data from Nullet and Juvik (1997).

Along an elevation gradient spanning 2600–3800 m we found a strong negative relationship (*R*^2^ = 0.96; *P* < 0.001 for linear regression model) between elevation and predawn adaxial T_UV A_ in the non-native *V. thapsus* but predawn T_UV A_ did not vary (*R*^2^ = 0.02; *P* = 0.87 for linear regression model) with elevation in the native shrub *V. reticulatum* (**Figure 3A**). However, *V. reticulatum* maintained 2-4 times higher predawn levels of UV screening (mean T_UV A_ = 2.8-3.1%) than *V. thapsus* (mean T_UV A_ = 6.0-11.2%) at similar elevations, based on estimates from regression models. For *V. thapsus*, predawn T_UV A_ exhibited a non-linear, negative relationship (*R*^2^ = 0.986; *P* = 0.003 for third-order polynomial regression model) with relative peak daily clear sky UV-B_ERY_ along this elevation gradient (**Figure 3B**). Similar relationships were found with daily maximum clear sky total SW irradiance (*R*^2^ = 0.982; *P* < 0.001 for a linear model; not shown) and PAR (*R*^2^ = 0.948; *P* = 0.003 for a second-order polynomial model; not shown) though the relative irradiance changes in these two wavebands over this sampling gradient were less (5% for PAR and 12% for SW) than that for UV-B_ERY_ (ca. 20%). There were no significant relationships between predawn T_UV A_ and these relative measures of solar radiation (UV-B_ERY_, PAR and SW) for *V. reticulatum* (*R*^2^ < 0.02; *P* > 0.8; not shown).

## Discussion

Results from our survey of 19 species representing 13 different plant families indicate that significant interspecific variation exists in maximum (midday) levels of UV screening for plants growing in the Mauna Kea alpine/upper subalpine. Overall, however, epidermal T_UV A_ was low for all taxa (means ranged from 2.6 to 11.5%). Further, we found no significant differences in UV screening between native and non-native species. Most of the native species in our study were woody dicots (five of the eight species sampled) whereas the majority of the non-native species were herbaceous dicots (9 of 11 species sampled). Therefore, our comparison of native vs. non-native species was, to some degree, confounded with growth form effects. Day et al. (1992) reported significantly greater leaf epidermal UV transmittance in herbaceous dicots (*n* = 7 species) than woody dicots (*n* = 3 species) for plants growing in a temperate subalpine meadow in Wyoming, United States (3310 m elevation). By comparison, we detected no significant effect of growth form on leaf optical properties in this tropical alpine environment. Our study was similar in size and scope to the study of Day et al. (1992) in that the majority (74%) of the species we tested were also herbaceous and most of these (71%) were forbs. Thus, even though differences in growth form composition existed between native and non-native species in our study, these differences appeared to have little effect on overall levels of UV screening between these two categories of species. It seems that growth form effects on T_UV A_ are less pronounced in the extreme UV-B conditions in the tropical alpine than in lower UV-B environments, which occur at lower elevations and higher latitudes.

Eldredge and Evenhuis (2003) reported that of the 2311 species of vascular plants known to occur in the Hawaiian Islands ca. 50% (1160) of these species are non-native (non-indigenous) in origin. Only a small fraction (73 species or ca. 3%) of these species occurs in the alpine/upper-subalpine of Mauna Kea though the relative floristic composition of non-native species in this life zone (54%) is comparable to that of the Hawaiian Islands in general (Gerrish, 2013). In our study, we examined about one-third of the native and non-native species of the Mauna Kea alpine/upper subalpine (8 native and 11 non-native species). Our results are therefore derived from sizable and comparable fractions of the native and non-native flora present in this habitat and there is no reason to believe that our findings would have differed had we sampled a greater number of species in the Mauna Kea alpine.

While it is seldom possible to determine the exact origin of non-native species, the non-native species included in our study clearly originated from lower elevation, higher latitude sites where UV-B exposure would be considerably less than our sampling locations. The majority of species (8 of 11) are listed in floras as originating from Europe or Eurasia. More detailed distributions of several species indicate they typically originate north of 30° latitude (Weber, 2017). The remaining species also originate outside the low-latitude tropics: *Heterotheca grandiflora* from coastal California (Munz, 1968), *O. stricta* from southern South America (Robberecht and Caldwell, 1983), and *S. madagascariensis* from South Africa (as determined by phylogenetic analysis; Le Roux et al., 2006). Whereas, it is possible that these species were pre-adapted to the extreme UV-B conditions in the tropical alpine, it seems more likely that they either (1) evolved higher UV screening over the course of their invasion and colonization of montane environments in Hawaii, or (2) that they possess high degrees of phenotypic plasticity in UV tolerance that then enabled them to acclimate to a wide range of UV-B conditions. Some of the non-native species in our study have arrived in Hawaii relatively recently and are highly invasive pests (e.g., *S. madagascariensis*) whereas others have been on the island sufficiently long enough to be considered “naturalized” (e.g., *P. pratensis* and *Rumex acetosella*) (Wagner et al., 1999; USDA, 2017). Thus, the non-native species in our study have experienced various periods of time since their introductions and these historical differences may have influenced the degree to which their adjustments in UV protection reflect genetic changes in populations vs. phenotypic plasticity.

In general, the success of non-native, invasive species is often attributed to their high levels of phenotypic plasticity, which then enables them to cope with a wide array of environmental conditions (Davidson et al., 2011). Many of the non-native species in our study, regardless of the timing of their introductions, may have achieved levels of UV protection that are similar to those of the native alpine species via phenotypic adjustment (i.e., acclimation) to the high UV-B irradiances in this alpine environment. In the case of *V. thapsus*, this is an herbaceous weed at many elevations in the temperate zone and this species has been found to exhibit a high degree of phenotypic plasticity rather than rapid evolution over the course of its invasions (Parker et al., 2003). Findings from the current study revealed that predawn T_UV A_ varied in a linear fashion (1.3% change in relative T_UV A_ per 100 m) with elevation in *V. thapsus* whereas this was not the case for the native *V. reticulatum* (**Figure 3A**). Using a similar approach, but conducting measurements at midday over a narrower elevation range (ca. 800 m), Ruhland et al. (2013) reported linear decreases in T_UV A_ with increasing elevation (9.7% per 100 m) for the native shrub *Artemisia tridentata* in Wyoming, United States. Because of the short distances between their sampling sites, these authors attributed the elevation variation in UV screening in *A. tridentata* to phenotypic plasticity rather than ecotypic differentiation. In a growth chamber study, Beckmann et al. (2012) found similar levels of phenotypic plasticity in native (German) and non-native (New Zealand) populations of *Hieracium pilosella* in morphological and growth responses to UV-B, though some genetic differentiation also occurred between these two populations. Thus, at present it is not clear whether non-native species (or populations) exhibit greater phenotypic plasticity in UV protection than native plants and further study on a greater number and diversity of species is needed to adequately test this hypothesis. It is conceivable, however, that phenotypic plasticity in UV protection in *V. thapsus* is one factor that has aided the invasion of this particular species in Hawaii.

In this study, we focused on the attenuation of incoming UV by the epidermis (i.e., UV screening) as this mechanism provides the first line of defense against the potentially deleterious effects of UV-B. However, UV protection in plants involves not only UV screening but other factors as well, such as levels of antioxidant compounds, DNA repair and leaf thickness, that all serve to protect and repair sensitive targets from direct and indirect UV-induced injury (Britt, 1999; Jacques et al., 2009; Majer et al., 2014; Robson et al., 2015). In some cases, these other mechanisms of UV protection have been shown to vary with elevation. For example, Wang Q. W. et al. (2016) found that differential sensitivity to UV radiation between high vs. low elevation populations and species of *Arabidopsis* growing in the Hakkado Mountains, Japan, was attributable, in part, to population differences in DNA damage and repair. Wildi and Lütz (1996) reported that total antioxidant levels increased with elevation in several species in the Austrian Alps, but whether these differences were due to elevation changes in UV, temperature, or other factors was not assessed. Moreover, while the attenuation of UV within the leaf is predominantly influenced by UV-absorbing compounds, the surface features of leaves (e.g., trichomes and waxes) can also influence leaf optical properties (Karabourniotis et al., 1992; Holmes and Keiller, 2002) and in some cases these traits have been shown to vary with elevation (e.g., Pilon et al., 1999). One of the species in our study (*V. thapsus*) possesses pubescent leaves and it is conceivable that the trichomes of this species are also involved in UV screening. We did not test whether these hairs absorb or reflect UV nor did we evaluate whether there were elevation changes in the levels of pubescence in this species. Increases in pubescence would add to the UV-filtering effect of the epidermis and thus further decrease T_UV A_ but only if the leaf hairs possessed UV-absorbing compounds. Pubescence would likely have no effect on T_UV A_, as measured with the UVA-PAM, if the hairs primarily reflect UV, as they would also reflect visible (including blue light) radiation (Holmes and Keiller, 2002). In this situation, the ratio of *F*_UV A_/*F*_BL_ and thus T_UV A_ would be unaffected by variation in trichome density.

Because of their known function in UV protection and potential value in plant systematics, a large number of studies have examined elevation changes in the levels of flavonoids (and related phenolic compounds) and/or UV screening in a variety of plant species (e.g., Caldwell et al., 1982; McDougal and Parks, 1984; Barnes et al., 1987; Rau and Hofmann, 1996; Alonso-Amelot et al., 2007; González et al., 2007; Rieger et al., 2008; Bernal et al., 2013; Ruhland et al., 2013; Cirak et al., 2017; and others). Some of these studies have further tested the linkage between elevation variation in flavonoids/UV screening and UV tolerance. In one of the most extensive studies to date, Sullivan et al. (1992) examined 33 species collected along a 3000 m elevation gradient in Maui, Hawaii, and found a significant, inverse relationship between elevation and negative effects of UV-B on growth for plants growing in a greenhouse. A companion study by Ziska et al. (1992) showed that greater sensitivity to UV-induced partial inhibition of photosynthesis in a subset of greenhouse-grown low elevation species (*n* = 4) was associated with lower constitutive levels of UV-absorbing compounds relative to high elevation taxa. Thus, even though our study only examined UV screening, our findings imply that non-native species would not be more or less prone to UV-induced injury than native species under the extreme UV-B conditions in this tropical alpine environment. Furthermore, our findings that non-native species possess levels of UV screening that are comparable to those of native species do not support the general expectation that non-native species invest more heavily into resource acquisition and growth at the expense of stress tolerance mechanisms than native species (Van Kleunen et al., 2010). Rather, it appears that the low resource, highly stressful environment of the alpine serves as a strong filter of plant species (Alexander et al., 2011; Gerrish, 2013) and functional traits (Funk et al., 2016) such that native and non-native species in this environment exhibit little difference in UV defense.

Elevation gradients, such as the one in our study, are complex gradients where a number of environmental factors (e.g., solar radiation, temperature, precipitation) change in concert (Körner, 2007). Studies such as ours linking elevation changes in UV screening to changes in UV-B are therefore, correlative at best, and other environmental factors may contribute to this variation. Indeed, it is well known that low temperatures can increase UV-absorbing compounds and UV screening in leaves (e.g., Bilger et al., 2007) and some have found elevation changes in flavonoids to be more strongly influenced by changes in temperature than UV (Albert et al., 2009). Nonetheless, studies along elevation gradients can provide insights into how plants might respond to the changes in UV-B that occurs with migration to higher elevations as a consequence of climate change. In our study, we found a strong negative relationship between clear sky erythemal UV-B and T_UV A_ in *V. thapsus*, but we also detected strong relationships with PAR and total SW radiation. In general, radiant fluxes of biologically effective UV-B increase proportionally more with elevation than those of UV-A, PAR or SW in temperate and tropical mountains (Caldwell et al., 1980; Piazena, 1996; Blumthaler et al., 1997; McKenzie et al., 2001). Such appears to be the case for this elevation gradient in Hawaii (Nullet and Juvik, 1997). Thus, while migration to higher elevations exposes plants to increases in solar radiation in all wavebands, the relative changes are greatest for biologically effective UV-B. These elevation gradients in UV-B can be further accentuated by the presence of clouds. For example, because of a persistent, dense cloud layer at ca. 2000 masl that results from trade-wind inversions, the differences in UV-B between the alpine and sea level differ considerably from the eastern, wind-ward side of Mauna Kea to the western, lee-ward side of the mountain. From continuous UV measurements, Nullet and Juvik (1997) reported that monthly erythemal UV-B, when averaged over all sky conditions, was actually 55–103% greater at 3400 masl than at a windward sea level location, depending on time of year (summer vs. winter), in comparison to the ca. 20% difference in clear sky UV-B between these elevations. Plant species that occur below the cloud layer on the moist, eastern side of Mauna Kea, and which migrate to elevations above this cloud layer would therefore likely require greater acclimation to UV-B than would those migrating comparable elevations on the drier, eastern side of this mountain.

## Conclusion

Our findings indicate that high levels of UV screening are not restricted to plant species native to the high UV-B conditions of the tropical alpine and that plasticity in epidermal UV transmittance is a mechanism employed by some, but not all, species to cope with varying solar UV exposures. Whether this plasticity in UV screening is a general feature of non-native species is unknown, but our findings do suggest that many terrestrial plants will be able to tolerate the increased levels of UV-B radiation as they migrate to higher elevations as a consequence of climate change.

## Author Contributions

PB and RR collected and analyzed the data. PB wrote the manuscript with the participation of SF. PB, RR, and SF designed the studies and all were involved in securing funding for the research. RR died before the final draft of the manuscript was completed but he assisted with the preparation of early drafts.

## Conflict of Interest Statement

The authors declare that the research was conducted in the absence of any commercial or financial relationships that could be construed as a potential conflict of interest.

## References

[B1] Agati G. Azzarello E. Pollastri S. Tattini M. (2012). Flavonoids as antioxidants in plants: location and functional significance. *Plant Sci.* 196 67–76. 10.1016/j.plantsci.2012.07.014 23017900

[B2] Albert A. Sareedenchai V. Heller W. Seidlitz H. K. Zidorn C. (2009). Temperature is the key to altitudinal variation of phenolics in *Arnica montana* L. cv. ARBO. *Oecologia* 160 1–8. 10.1007/s00442-009-1277-1 19194724

[B3] Albert K. R. Mikkelsen T. N. Ro-Poulsen H. Arndal M. F. Michelsen A. (2011). Ambient UV-B radiation reduces PSII performance and net photosynthesis in high Arctic *Salix arctica*. *Environ. Exp. Bot.* 73 10–18. 10.1016/j.envexpbot.2011.08.003

[B4] Alexander J. M. Kueffer C. Daehler C. C. Edwards P. J. Pauchard A. Seipel T. (2011). Assembly of nonnative floras along elevational gradients explained by directional ecological filtering. *Proc. Natl. Acad. Sci. U.S.A.* 108 656–661. 10.1073/pnas.1013136108: 21187380 PMC3021079

[B5] Alonso-Amelot M. E. Oliveros-Bastidas A. Calcagno-Pisarelli M. P. (2007). Phenolics and condensed tannins of high altitude *Pteridium arachnoideum* in relation to sunlight exposure, elevation, and rain regime. *Biochem. Syst. Ecol.* 35 1–10. 10.1016/j.bse.2006.04.013

[B6] Averett J. P. McCune B. Parks C. G. Naylor B. J. DelCurto T. Mata-Gonzalez R. (2016). Non-native plant invasion along elevation and canopy closure gradients in a middle Rocky Mountain ecosystem. *PLoS ONE* 11:e0147826 10.1371/journal.pone.0147826 PMC4732764 26824750

[B7] Ballaré C. L. Caldwell M. M. Flint S. D. Robinson S. A. Bornman J. F. (2011). Effects of solar ultraviolet radiation on terrestrial ecosystems. Patterns, mechanisms, and interactions with climate change. *Photochem. Photobiol. Sci.* 10 226–241. 10.1039/c0pp90035d 21253661

[B8] Bandurska H. Niedziela J. Chadzinikolau T. (2013). Separate and combined responses to water deficit and UV-B radiation. *Plant Sci.* 213 98–105. 10.1016/j.plantsci.2013.09.003 24157212

[B9] Barnes P. W. Flint S. D. Caldwell M. M. (1987). Photosynthesis damage and protective pigments in plants from a latitudinal arctic/alpine gradient exposed to supplemental UV-B radiation in the field. *Arctic Alpine Res.* 19 21–27. 10.2307/1550996

[B10] Barnes P. W. Flint S. D. Ryel R. J. Tobler M. A. Barkley A. E. Wargent J. J. (2015). Rediscovering leaf optical properties: new insights into plant acclimation to solar UV radiation. *Plant Physiol. Biochem.* 93 94–100. 10.1016/j.plaphy.2014.11.015 25465528

[B11] Barnes P. W. Flint S. D. Slusser J. R. Gao W. Ryel R. J. (2008). Diurnal changes in epidermal UV transmittance of plants in naturally high UV environments. *Physiol. Plant.* 133 363–372. 10.1111/j.1399-3054.2008.01084.x 18346077

[B12] Barnes P. W. Flint S. D. Tobler M. A. Ryel R. J. (2016a). Diurnal adjustment in UV-sunscreen protection is widespread among higher plants. *Oecologia* 181 55–63. 10.1007/s00442-016-3558-9 26809621

[B13] Barnes P. W. Searles P. S. Ballaré C. L. Ryel R. J. Caldwell M. M. (2000). Non-invasive measurements of leaf epidermal transmittance of UV radiation using chlorophyll fluorescence: field and laboratory studies. *Physiol. Plant.* 109 274–283. 10.1034/j.1399-3054.2000.100308.x

[B14] Barnes P. W. Tobler M. A. Keefover-Ring K. Flint S. D. Barkley A. E. Ryel R. J. (2016b). Rapid modulation of ultraviolet shielding in plants is influenced by solar ultraviolet radiation and linked to alterations in flavonoids. *Plant Cell Environ.* 39 222–230. 10.1111/pce.12609 26177782

[B15] Beckmann M. Hock M. Bruelheide H. Erfmeier A. (2012). The role of UV-B radiation in the invasion of *Hieracium pilosella*–a comparison of German and New Zealand plants. *Environ. Exp. Bot.* 75 173–180. 10.1016/j.envexpbot.2011.09.010

[B16] Benavides R. Escudero A. Coll L. Ferrandis P. Ogaya R. Gouriveau F. (2016). Recruitment patterns of four tree species along elevation gradients in Mediterranean mountains: not only climate matters. *For. Ecol. Manage.* 360 287–296. 10.1016/j.foreco.2015.10.043

[B17] Bernal M. Llorens L. Julkunen-Tiitto R. Badosa J. Verdaguer D. (2013). Altitudinal and seasonal changes of phenolic compounds in *Buxus sempervirens* leaves and cuticles. *Plant Physiol. Biochem.* 70 471–482. 10.1016/j.plaphy.2013.06.012 23845826

[B18] Bidel L. P. R. Meyer S. Goulas Y. Cadot Y. Cerovic Z. G. (2007). Responses of epidermal phenolic compounds to light acclimation: in vivo qualitative and quantitative assessment using chlorophyll fluorescence excitation spectra in leaves of three woody species. *J. Photochem. Photobiol. B Biol.* 88 163–179. 10.1016/j.jphotobiol.2007.06.002 17720509

[B19] Bilger W. Johnsen T. Schreiber U. (2001). UV-excited chlorophyll fluorescence as a tool for the assessment of UV-protection by the epidermis of plants. *J. Exp. Bot.* 52 2007–2014. 10.1093/jexbot/52.363.2007 11559736

[B20] Bilger W. Rolland M. Nybakken L. (2007). UV screening in higher plants induced by low temperature in the absence of UV-B radiation. *Photochem. Photobiol. Sci.* 6 190–195. 10.1039/B609820G 17277843

[B21] Blumthaler M. Ambach W. Ellinger R. (1997). Increase in solar UV radiation with altitude. *J. Photochem. Photobiol. B Biol.* 39 130–134. 10.1016/S1011-1344(96)00018-8

[B22] Bornman J. F. Barnes P. W. Robinson S. A. Ballaré C. L. Flint S. D. Caldwell M. M. (2015). Solar ultraviolet radiation and ozone depletion-driven climate change: effects on terrestrial ecosystems. *Photochem. Photobiol. Sci.* 14 88–107. 10.1039/c4pp90034k 25435216

[B23] Britt A. B. (1999). Molecular genetics of DNA repair in higher plants. *Trends Plant Sci.* 4 20–25. 10.1016/S1360-1385(98)01355-7 10234266

[B24] Brock M. T. Galen C. (2005). Drought tolerance in the alpine dandelion, *Taraxacum ceratophorum* (Asteraceae), its exotic congener *T. officinale*, and interspecific hybrids under natural and experimental conditions. *Am. J. Bot.* 92 1311–1321. 10.3732/ajb.92.8.1311 21646151

[B25] Caldwell M. M. Robberecht R. Billings W. D. (1980). A steep latitudinal gradient of solar ultraviolet-B radiation in the arctic-alpine life zone. *Ecology* 61 600–611. 10.2307/1937426

[B26] Caldwell M. M. Robberecht R. Flint S. D. (1983). Internal filters: prospects for UV-acclimation in higher plants. *Physiol. Plant.* 58 445–450. 10.1111/j.1399-3054.1983.tb04206.x

[B27] Caldwell M. M. Robberecht R. Nowak R. S. Billings W. D. (1982). Differential photosynthetic inhibition by ultraviolet radiation in species from the arctic-alpine life zone. *Arctic Alpine Res.* 14 195–202. 10.2307/1551152

[B28] Chapin F. S. Körner C. (1994). Arctic and alpine biodiversity: patterns, causes and ecosystem consequences. *Trends Ecol. Evol.* 9 45–47. 10.1016/0169-5347(94)90266-6 21236764

[B29] Cirak C. Radusiene J. Jakstas V. Ivanauskas L. Seyis F. Yayla F. (2017). Altitudinal changes in secondary metabolite contents of *Hypericum androsaemum* and *Hypericum polyphyllum*. *Biochem. Syst. Ecol.* 70 108–115. 10.1016/j.bse.2016.11.006

[B30] Cuyckens G. A. E. Christie D. A. Domic A. I. Malizia L. R. Renison D. (2016). Climate change and the distribution and conservation of the world’s highest elevation woodlands in the South American Altiplano. *Glob. Planet. Change* 137 79–87. 10.1016/j.gloplacha.2015.12.010

[B31] Dainese M. Alkio S. Hulme P. E. Bertolli A. Prosser F. Marini L. (2017). Human disturbance and upward expansion of plants in a warming climate. *Nat. Clim. Change* 10.1038/nclimate3337 [Epub ahead of print].

[B32] Davidson A. M. Jennions M. Nicotra A. B. (2011). Do invasive species show higher phenotypic plasticity than native species and, if so, is it adaptive? A meta-analysis. *Ecol. Lett.* 14 419–431. 10.1111/j.1461-0248.2011.01596.x 21314880

[B33] Day T. A. Martin G. Vogelmann T. C. (1993). Penetration of UV-B radiation in foliage: evidence that the epidermis behaves as a non-uniform filter. *Plant Cell Environ.* 16 735–741. 10.1111/j.1365-3040.1993.tb00493.x

[B34] Day T. A. Vogelmann T. C. DeLucia E. H. (1992). Are some plant life forms more effective than others in screening out ultraviolet-B radiation? *Oecologia* 92 513–519. 10.1007/BF00317843 28313222

[B35] Dolezal J. Dvorsky M. Kopecky M. Liancourt P. Hiiesalu I. Macek M. (2016). Vegetation dynamics at the upper elevational limit of vascular plants in Himalaya. *Sci. Rep.* 6:24881 10.1038/srep24881 PMC4855180 27143226

[B36] Eldredge L. G. Evenhuis N. L. (2003). Hawaii’s biodiversity: a detailed assessment of the numbers of species in the Hawaiian Islands. *Bishop Mus. Occas. Pap.* 76 1–28.

[B37] Flint S. D. Searles P. S. Caldwell M. M. (2004). Field testing of biological spectral weighting functions for induction of UV-absorbing compounds in higher plants. *Photochem. Photobiol.* 79 399–403. 10.1111/j.1751-1097.2004.tb00026.x 15191047

[B38] Funk J. L. (2013). The physiology of invasive plants in low-resource environments. *Conserv. Physiol.* 1:cot026 10.1093/conphys/cot026 PMC4806624 27293610

[B39] Funk J. L. Standish R. J. Stock W. D. Valladares F. (2016). Plant functional traits of dominant native and invasive species in mediterranean-climate ecosystems. *Ecology* 97 75–83. 10.1890/15-0974.1 27008777

[B40] Gerrish G. (2013). *Botanical Baseline Survey* (2011) of the University of Hawaii’s Managed Lands on Mauna Kea. Hilo, HI: University of Hawaii-Hilo.

[B41] González J. A. Gallardo M. G. Boero C. Liberman Cruz M. Prado F. E. (2007). Altitudinal and seasonal variation of protective and photosynthetic pigments in leaves of the world’s highest elevation trees *Polylepis tarapacana* (Rosaceae). *Acta Oecol.* 32 36–41. 10.1016/j.actao.2007.03.002

[B42] Hideg E. Jansen M. A. K. Strid A. (2013). UV-B exposure, ROS, and stress: inseparable companions or loosely linked associates? *Trends Plant Sci.* 18 107–115. 10.1016/j.tplants.2012.09.003 23084465

[B43] Hofmann R. W. Jahufer M. Z. Z. (2011). Tradeoff between biomass and flavonoid accumulation in white clover reflects contrasting plant strategies. *PLoS ONE* 6:e18949 10.1371/journal.pone.0018949 PMC3079752 21526153

[B44] Holmes M. G. Keiller D. R. (2002). Effects of pubescence and waxes on the reflectance of leaves in the ultraviolet and photosynthetic wavebands: a comparison of a range of species. *Plant Cell Environ.* 25 85–93. 10.1046/j.1365-3040.2002.00779.x

[B45] IPCC (2014). *Climate Change 2014: Impacts, Adaptation, and Vulnerability. Part A: Global and Sectoral Aspects. Contribution of Working Group II to the Fifth Assessment Report of the Intergovernmental Panel on Climate Change* eds Field C. B. Barros V. R. Dokken D. J. Mach K. J. Mastrandrea M. D. Bilir T. E. Cambridge: Cambridge University Press.

[B46] Jacques E. Hectors K. Guisez Y. Verbelen J. P. Vissenberg K. Prinsen E. (2009). Leaf and cell development during UV-B acclimation in *Arabidopsis thaliana*. *Comp. Biochem. Physiol. A* 153 S203–S203. 10.1016/j.cbpa.2009.04.639

[B47] Jansen M. A. K. Bornman J. F. (2012). UV-B radiation: from generic stressor to specific regulator. *Physiol. Plant.* 145 501–504. 10.1111/j.1399-3054.2012.01656.x 22646504

[B48] Jansen M. A. K. Gaba V. Greenberg B. M. (1998). Higher plants and UV-B radiation: balancing damage, repair and acclimation. *Trends Plant Sci.* 3 131–135. 10.1016/S1360-1385(98)01215-1

[B49] Jenkins G. I. (2014). The UV-B photoreceptor UVR8: from structure to physiology. *Plant Cell* 26 21–37. 10.1105/tpc.113.119446 24481075 PMC3963570

[B50] Julkunen-Tiitto R. Nenadis N. Neugart S. Robson M. Agati G. Vepsäläinen J. (2015). Assessing the response of plant flavonoids to UV radiation: an overview of appropriate techniques. *Phytochem. Rev.* 14 273–297. 10.1007/s11101-014-9362-4

[B51] Karabourniotis G. Papadopoulos K. Papamarkou M. Manetas Y. (1992). Ultraviolet-B radiation absorbing capacity of leaf hairs. *Physiol. Plant.* 86 414–418. 10.1111/j.1399-3054.1992.tb01337.x

[B52] Kolb C. A. Schreiber U. Gademann R. Pfündel E. E. (2005). UV-A screening in plants determined using a new portable fluorimeter. *Photosynthetica* 43 371–377. 10.1007/s11099-005-0061-7

[B53] Körner C. (2003). *Alpine Plant Life: Functional Plant Ecology of High Mountain Ecosystems.* New York, NY: Springer-Verlag 10.1007/978-3-642-18970-8

[B54] Körner C. (2007). The use of ‘altitude’ in ecological research. *Trends Ecol. Evol.* 22 569–574. 10.1016/j.tree.2007.09.006 17988759

[B55] Le Roux J. J. Wieczorek A. M. Ramadan M. M. Tran C. T. (2006). Resolving the native provenance of invasive fireweed (*Senecio madagascariensis* Poir.) in the Hawaiian Islands as inferred from phylogenetic analysis. *Divers. Distrib.* 12 694–702. 10.1111/j.1472-4642.2006.00271.x

[B56] Lee D. W. Lowry J. B. (1980). Solar ultraviolet on tropical mountains: Can it affect plant speciation? *Am. Nat.* 115 880–882. 10.1086/283606

[B57] Majer P. Neugart S. Krumbein A. Schreiner M. Hideg E. (2014). Singlet oxygen scavenging by leaf flavonoids contributes to sunlight acclimation in *Tilia platyphyllos*. *Environ. Exp. Bot.* 100 1–9. 10.1016/j.envexpbot.2013.12.001

[B58] Markstadter C. Queck I. Baumeister J. Riederer M. Schreiber U. Bilger W. (2001). Epidermal transmittance of leaves of *Vicia faba* for UV radiation as determined by two different methods. *Photosynth. Res.* 67 17–25. 10.1023/A:1010676111026 16228313

[B59] Mazza C. A. Boccalandro H. E. Giordano C. V. Battista D. Scopel A. L. Ballaré C. L. (2000). Functional significance and induction by solar radiation of ultraviolet-absorbing sunscreens in field-grown soybean crops. *Plant Physiol.* 122 117–125. 10.1104/pp.122.1.117 10631255 PMC58850

[B60] McDougal K. M. Parks C. R. (1984). Elevational variation in foliar flavonoids of *Quercus rubra* L. (Fagaceae). *Am. J. Bot.* 71 301–308. 10.2307/2443490

[B61] McKenzie R. L. Johnston P. V. Smale D. Bodhaine B. A. Madronich S. (2001). Altitude effects on UV spectral irradiance deduced from measurements at Lauder, New Zealand, and at Mauna Loa Observatory, Hawaii. *J. Geophys. Res.* 106 22845–22860. 10.1029/2001jd900135

[B62] Mewis I. Schreiner M. Chau Nhi N. Krumbein A. Ulrichs C. Lohse M. (2012). UV-B irradiation changes specifically the secondary metabolite profile in broccoli sprouts: induced signaling overlaps with defense response to biotic stressors. *Plant Cell Physiol.* 53 1546–1560. 10.1093/pcp/pcs096 22773681 PMC3439869

[B63] Morales L. O. Brosché M. Vainonen J. Jenkins G. I. Wargent J. J. Sipari N. (2013). Multiple roles for UV RESISTANCE LOCUS8 in regulating gene expression and metabolite accumulation in *Arabidopsis* under solar ultraviolet radiation. *Plant Physiol.* 161 744–759. 10.1104/pp.112.211375 23250626 PMC3561016

[B64] Munz P. A. (1968). *Supplement to a California Flora.* Berkeley, CA: University of California Press.

[B65] Nullet D. Juvik J. O. (1997). Measured altitudinal profiles of UV-B iradiance in Hawai’i. *Phys. Geogr.* 18 335–345.

[B66] Nybakken L. Aubert S. Bilger W. (2004). Epidermal UV-screening of arctic and alpine plants along a latitudinal gradient in Europe. *Polar Biol.* 27 391–398. 10.1007/s00300-004-0601-9

[B67] Palmer D. D. (2003). *Hawai_i’s Ferns and Fern Allies.* Honolulu, HI: University of Hawaii Press.

[B68] Parker I. M. Rodriguez J. Loik M. E. (2003). An evolutionary approach to understanding the biology of invasions: local adaptation and general-purpose genotypes in the weed *Verbascum thapsus*. *Conserv. Biol.* 17 59–72. 10.1046/j.1523-1739.2003.02019.x

[B69] Pfündel E. E. Ben Ghozlen N. Meyer S. Cerovic Z. G. (2007). Investigating UV screening in leaves by two different types of portable UV fluorimeter reveals in vivo screening by anthocyanins and carotenoids. *Photosynth. Res.* 93 205–221. 10.1007/s11120-007-9135-7 17286190

[B70] Piazena H. (1996). The effect of altitude upon the solar UV-B and UV-A irradiance in the tropical Chilean Andes. *Solar Energy* 57 133–140. 10.1016/S0038-092X(96)00049-7

[B71] Pilon J. J. Lambers H. Baas W. Tosserams M. Rozema J. Atkin O. K. (1999). Leaf waxes of slow-growing alpine and fast-growing lowland *Poa* species: inherent differences and responses to UV-B radiation. *Phytochemistry* 50 571–580. 10.1016/S0031-9422(98)00556-1

[B72] Pyšek P. Jarošík V. Pergl J. Wild J. (2011). Colonization of high altitudes by alien plants over the last two centuries. *Proc. Natl. Acad. Sci. U.S.A.* 108 439–440. 10.1073/pnas.1017682108 21189300 PMC3021013

[B73] Qi Y. Heisler G. M. Gao W. Vogelmann T. C. Bai S. (2010). “Characteristics of UV-B radiation tolerance in broadleaf trees in southern United States,” in *UV Radiation in Global Climate Change. Measurements, Modeling and Effects on Ecosystems* eds Gao W. Schmoldt D. L. Slusser J. R. (Berlin: Springer-Verlag) 509–530.

[B74] Randriamanana T. R. Nissinen K. Moilanen J. Nybakken L. Julkunen-Tiitio R. (2015). Long-term UV-B and temperature enhancements suggest that females of *Salix myrsinifolia* plants are more tolerant to UV-B than males. *Environ. Exp. Bot.* 109 296–305. 10.1016/j.envexpbot.2014.06.007

[B75] Rau W. Hofmann H. (1996). Sensitivity to UV-B of plants growing in different altitudes in the Alps. *J. Plant Physiol.* 148 21–25. 10.1016/S0176-1617(96)80289-6

[B76] Richards C. L. Bossdorf O. Muth N. Z. Gurevitch J. Pigliucci M. (2006). Jack of all trades, master of some? On the role of phenotypic plasticity in plant invasions. *Ecol. Lett.* 9 981–993. 10.1111/j.1461-0248.2006.00950.x 16913942

[B77] Rieger G. Müller M. Guttenberger H. Bucar F. (2008). Influence of altitudinal variation on the content of phenolic compounds in wild populations of *Calluna vulgaris, Sambucus nigra*, and *Vaccinium myrtillus*. *J. Agric. Food Chem.* 56 9080–9086. 10.1021/jf801104e 18788745

[B78] Robberecht R. Caldwell M. M. (1983). Protective mechanisms and acclimation to solar ultraviolet-B radiation in *Oenothera stricta*. *Plant Cell Environ.* 6 477–485. 10.1111/1365-3040.ep11588121

[B79] Robberecht R. Caldwell M. M. Billings W. (1980). Leaf ultraviolet optical properties along a latitudinal gradient in the arctic-alpine life zone. *Ecology* 61 612–619. 10.2307/1937427

[B80] Robson T. M. Klem K. Urban O. Jansen M. A. K. (2015). Re-interpreting plant morphological responses to UV-B radiation. *Plant Cell Environ.* 38 856–866. 10.1111/pce.12374 24890713

[B81] Rozema J. Chardonnens A. Tosserams M. Hafkenscheid R. Bruijnzeel S. (1997). Leaf thickness and UV-B absorbing pigments of plants in relation to an elevational gradient along the Blue Mountains, Jamaica. *Plant Ecol.* 128 150–159. 10.1023/A:1009719109153

[B82] Ruhland C. T. Dyslin M. J. Krenz J. D. (2013). Wyoming big sagebrush screens ultraviolet radiation more effectively at higher elevations. *J. Arid Environ.* 96 19–22. 10.1016/j.jaridenv.2013.04.005

[B83] Savage J. Vellend M. (2015). Elevational shifts, biotic homogenization and time lags in vegetation change during 40 years of climate warming. *Ecography* 38 546–555. 10.1111/ecog.01131

[B84] Schreiner M. Mewis I. Huyskens-Keil S. Jansen M. A. K. Zrenner R. Winkler J. B. (2012). UV-B-induced secondary plant metabolites - potential benefits for plant and human health. *Crit. Rev. Plant Sci.* 31 229–240. 10.1080/07352689.2012.664979

[B85] Searles P. S. Flint S. D. Caldwell M. M. (2001). A meta-analysis of plant field studies simulating stratospheric ozone depletion. *Oecologia* 127 1–10. 10.1007/s004420000592 28547159

[B86] Siipola S. M. Kotilainen T. Sipari N. Morales L. O. Lindfors A. V. Robson T. M. (2015). Epidermal UV-A absorbance and whole-leaf flavonoid composition in pea respond more to solar blue light than to solar UV radiation. *Plant Cell Environ.* 38 941–952. 10.1111/pce.12403 25040832

[B87] Snell K. R. S. Kokubun T. Griffiths H. Convey P. Hodgson D. A. Newsham K. K. (2009). Quantifying the metabolic cost to an Antarctic liverwort of responding to an abrupt increase in UVB radiation exposure. *Glob. Change Biol.* 15 2563–2573. 10.1111/j.1365-2486.2009.01929.x

[B88] Suding K. N. Farrer E. C. King A. J. Kueppers L. Spasojevic M. J. (2015). Vegetation change at high elevation: scale dependence and interactive effects on Niwot Ridge. *Plant Ecol. Divers.* 8 713–725. 10.1080/17550874.2015.1010189

[B89] Sullivan J. H. Teramura A. H. Ziska L. H. (1992). Variation in UV-B sensitivity in plants from a 3,000-m elevational gradient in Hawaii. *Am. J. Bot.* 79 737–743. 10.2307/2444938

[B90] Tilbrook K. Arongaus A. B. Binkert M. Heijde M. Yin R. Ulm R. (2013). The UVR8 UV-B photoreceptor: perception, signaling and response. *Arabidopsis Book* 11:0164 10.1199/tab.0164 PMC3711356 23864838

[B91] USDA (2017). *The PLANTS Database.* Available at: http://plants.usda.gov [accessed April 5, 2017].

[B92] Van Kleunen M. Weber E. Fischer M. (2010). A meta-analysis of trait differences between invasive and non-invasive plant species. *Ecol. Lett.* 13 235–245. 10.1111/j.1461-0248.2009.01418.x 20002494

[B93] Wagner W. L. Herbst D. R. Khan N. Flynn T. (2012). *Hawaiian Vascular Plant Updates: A Supplement to the Manual the Flowering Plants of Hawai’i and Hawai’i’s ferns and fern allies.* Washington, DC: Smithsonian National Museum of Natural History.

[B94] Wagner W. L. Herbst D. R. Sohmer S. H. (1999). *Manual of the Flowering Plants of Hawai’i* Vols. 1 and 2 Honolulu, HI: University of Hawaii.

[B95] Wang H. Ma X. C. Zhang L. Siemann E. Zou J. W. (2016). UV-B has larger negative impacts on invasive populations of *Triadica sebifera* but ozone impacts do not vary. *J. Plant Ecol.* 9 61–68. 10.1093/jpe/rtv045

[B96] Wang Q. W. Kamiyama C. Hidema J. Hikosaka K. (2016). Ultraviolet-B-induced DNA damage and ultraviolet-B tolerance mechanisms in species with different functional groups coexisting in subalpine moorlands. *Oecologia* 181 1069–1082. 10.1007/s00442-016-3644-z 27139425

[B97] Wargent J. J. Nelson B. C. W. McGhie T. K. Barnes P. W. (2015). Acclimation to UV-B radiation and visible light in *Lactuca sativa* involves up-regulation of photosynthetic performance and orchestration of metabolome-wide responses. *Plant Cell Environ.* 38 929–940. 10.1111/pce.12392 24945714

[B98] Weber E. (2017). *Invasive Plant Species of the World: A Reference Guide to Environmental Weeds.* Oxfordshire: CABI.

[B99] Wildi B. Lütz C. (1996). Antioxidant composition of selected high alpine plant species from different altitudes. *Plant Cell Environ.* 19 138–146. 10.1111/j.1365-3040.1996.tb00235.x

[B100] Wolf A. Zimmerman N. B. Anderegg W. R. L. Busby P. E. Christensen J. (2016). Altitudinal shifts of the native and introduced flora of California in the context of 20th-century warming. *Glob. Ecol. Biogeogr.* 25 418–429. 10.1111/geb.12423

[B101] Zar J. H. (1999). *Biostatistical Analysis.* London: Pearson Education.

[B102] Zavala J. A. Mazza C. A. Dillon F. M. Chludil H. D. Ballaré C. L. (2015). Soybean resistance to stink bugs (*Nezara viridula* and *Piezodorus guildinii*) increases with exposure to solar UV-B radiation and correlates with isoflavonoid content in pods under field conditions. *Plant Cell Environ.* 38 920–928. 10.1111/pce.12368 24811566

[B103] Ziska L. H. Teramura A. H. Sullivan J. H. (1992). Physiological sensitivity of plants along an elevational gradient to UV-B radiation. *Am. J. Bot.* 79 863–871. 10.2307/2444995

